# Zeolite Properties, Methods of Synthesis, and Selected Applications

**DOI:** 10.3390/molecules29051069

**Published:** 2024-02-29

**Authors:** Natalia Kordala, Mirosław Wyszkowski

**Affiliations:** Department of Agricultural and Environmental Chemistry, University of Warmia and Mazury in Olsztyn, Łódzki 4 Sq., 10-727 Olsztyn, Poland; natalia.kordala@uwm.edu.pl

**Keywords:** zeolites, chemical synthesis, agriculture, environmental protection

## Abstract

Zeolites, a group of minerals with unique properties, have been known for more than 250 years. However, it was the development of methods for hydrothermal synthesis of zeolites and their large-scale industrial applications (oil processing, agriculture, production of detergents and building materials, water treatment processes, etc.) that made them one of the most important materials of the 20th century, with great practical and research significance. The orderly, homogeneous crystalline and porous structure of zeolites, their susceptibility to various modifications, and their useful physicochemical properties contribute to the continuous expansion of their practical applications in both large-volume processes (ion exchange, adsorption, separation of mixture components, catalysis) and specialized ones (sensors). The following review of the knowledge available in the literature on zeolites aims to present the most important information on the properties, synthesis methods, and selected applications of this group of aluminosilicates. Special attention is given to the use of zeolites in agriculture and environmental protection.

## 1. Introduction

**Figure 1:**
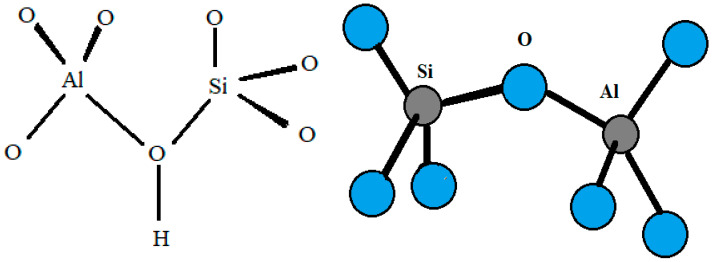
Scheme of silicon and aluminum tetrahedra in the zeolite structure (own elaboration based on Khaleque et al. [9]).

Zeolites are porous hydrated aluminosilicates with a three-dimensional structure containing cations of alkaline elements (sodium, potassium), alkaline earth (calcium, magnesium, less frequently barium, and strontium), or other monovalent or multivalent metals [1]. Due to their differentiated structure, which contains large free spaces and channels, zeolites exhibit properties characteristic of nanoporous materials and show the ability to lose and absorb water in amounts greater than 30% of their dry weight [2]. Zeolites have an ordered crystalline structure whose primary building units (PBU) are silicon [SiO_4_] and aluminum [AlO_4_] tetrahedra connected by common oxygen atoms [3], forming so-called secondary building units (SBU). According to Löwenstein’s rule, silicon–oxygen tetrahedra can be adjacent to each other (Si–O–Si), while aluminum–oxygen tetrahedra can only be connected to silicon–oxygen tetrahedra (Si–O–Al) [4]. Replacement of the Si^4+^ cation in the tetrahedral position by Al^3+^ results in an excess of electrons, i.e., a negative charge, which is usually compensated by so-called exchangeable cations (e.g., Na^+^, K^+^, NH_4_^+^, H^+^, Ca^2+^, Sr^2+^, or Mg^2+^) [5]. These off-grid cations, together with water molecules, are located in the free spaces of the aluminosilicate skeleton, moving freely inside the mineral and easily exchanging with other ions present in the environment [6]. The peculiar internal structure of zeolites is the result of a diverse distribution of tetrahedra, forming a network of structural chambers and channels of different sizes that, under normal temperature conditions, are filled with water molecules, the so-called zeolitic water [7]. By means of thermal treatment, this water can be easily removed without disturbing the crystal structure of the zeolite (Figure 1). The released pores can be filled with water molecules or other adsorbates [5,8].

**Figure 2:**
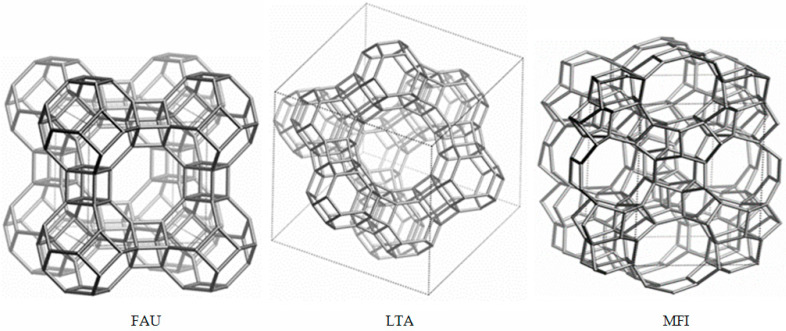
Elemental cell and channel system of FAU, LTA, and MFI zeolites (from Database of Zeolite Structures, International Zeolite Association [10]).

An example of an elementary cell and channel system of FAU, LTA, and MFI zeolites is shown in Figure 2.

The first zeolite (stilbite) was discovered in 1756 by the Swedish mineralogist Axel Frederic von Cronstedt [11,12]. The genesis of zeolite formation in nature is the reaction of volcanic rocks and ashes with water of high pH and high salt concentration [13]. Currently, we can distinguish about 50 natural zeolites, the most important of which are clinoptilolite, analcime, mordenite, and chabazite [14], and more than 150 synthetic zeolites [2]. Zeolites, both natural and synthetic, are used in various fields of human activity. On a larger scale, synthetic zeolites are mainly used because natural zeolites often contain various types of impurities, such as other minerals or metals [12,15]. In addition, synthetic zeolites tend to have better chemical and physical properties than natural zeolites [16,17]. The advantage of synthetic zeolites over natural zeolite applications is related to their greater stability in the reaction environment [18], as well as the pore size, which is larger in synthetic zeolites and allows adsorption of larger molecules (e.g., diesel oil) [13,18]. The kinetics of the removal of radioactive contaminants and heavy metal ions from synthetic materials are also several times higher compared to natural zeolites [9].

The aim of this paper is to present the sources in the natural environment as well as the properties and methods of synthesis of zeolites. The possibilities of using zeolites, mainly in agriculture and environmental protection, are also presented.

## 2. Properties and Classification of Zeolites

The presence of channels and chambers in the skeletal structure of zeolite gives it a number of desirable physicochemical properties and makes it a material with a wide range of applications. Zeolites have surface-active centers of acid–base or oxidation–reduction character. These are responsible for their exceptional adsorption and catalytic activity [19,20]. A characteristic feature of zeolites is the presence of micropores with diameters in the range of 0.3 to 1.0 nm [12] and a volume of micropores in the range of 0.10 to 0.35 cm^3^ g^−1^ [21]. The classification of zeolites based on the diameter of their pores is shown in Table 1.

**Figure 3:**
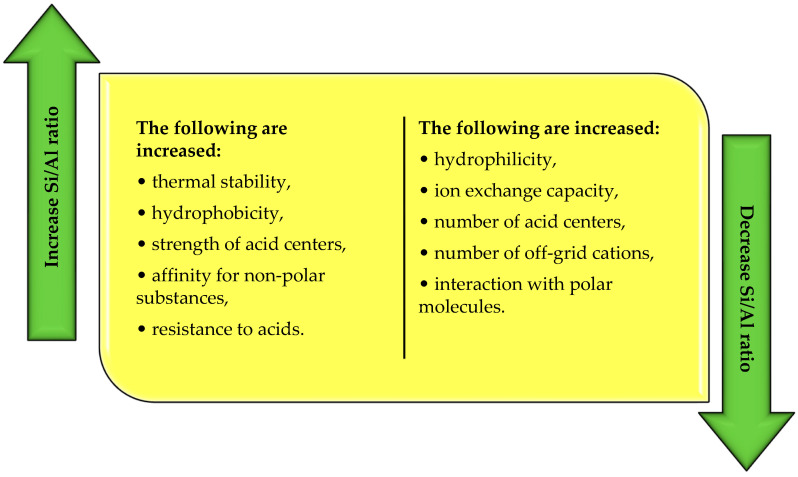
Changes in the physicochemical properties of zeolites as a function of the molar ratio of silicon to aluminum (own elaboration based on Payra, Dutta [24]; Jakubowski et al. [31]).

Another classification of zeolites concerns the molar ratio of Si/Al. According to Szostak [22], this ratio determines the physicochemical properties of zeolites (Figure 3). Based on the value of the Si/Al ratio, zeolites with low, medium, and high silicon content in the structure are distinguished (Table 2).

As the Si/Al ratio increases, the thermal stability of the zeolite structure, i.e., the resistance to amorphization or dealumination, increases. This relationship means that the structure of low-silicon zeolites can be affected as early as 700 °C, while the stability of high-silicon zeolites is preserved up to 1300 °C [21,23]. Low-silicon zeolites are also characterized by increased ion exchange capacity and hydrophilicity. High-silicon zeolites, on the other hand, are more hydrophobic and are characterized by an increased power of active centers, which predestines them for catalytic applications [13,18]. As the Si/Al ratio increases, the acidity also increases. On the other hand, under the same conditions, the amount of off-grid exchangeable cations in the structure and, consequently, the ion exchange capacity, which is proportional to the number of AlO_4_^−^ tetrahedra present in the zeolite skeleton, decreases [24].

The specific structure of zeolites gives them a number of unique properties [18]. They are good sorbents for water and adsorbents for uncharged molecules, effective ion exchangers and molecular sieves [25], and environmentally friendly catalysts [26]. Other important properties include the large internal surface area of the zeolite framework (several hundred m^2^ g^−1^) [13] and the cation exchange capacity, which varies between 200 and 300 cmol(+) kg^−1^ [27]. In addition, zeolites are characterized by low crystal density (from 1.9 to 2.2 Mg m^−3^) and low bulk density (e.g., 0.8 to 1.5 Mg m^−3^) [27]. Due to their unique physicochemical properties, zeolites have found applications in many industries, including environmental protection and agriculture [28,29].

**Table 2:** Classification of zeolites in terms of Si/Al ratio values (own elaboration based on Guisnet, Gilson [23]; Payra, Dutta [24]; Sharma et al. [30]).

Type of Zeolite	Si/Al Ratio	Example of Zeolite
Low silicon	1.0–1.5	4A, X, UZM-4, UZM-5
Medium silicon	~2.0–5.0	mordenite, zeolite Y, L
High silicon	>10	Beta, ZSM-5, ZSM-12
Silica molecular sieves	>100	silicites

## 3. Zeolite Synthesis Methods

Interest in synthetic zeolites has increased as newer possibilities for their application in various industrial fields have been discovered. Modern synthesis techniques make it possible to obtain zeolite material with specific parameters that can be modeled and adapted for different applications. In the zeolite process, the starting materials are usually silica and clay minerals, which are sources of aluminum and silicon. The reactants can also be waste materials such as red sludge, glass pellets, or fly ash [6], which show considerable similarity to zeolites in terms of chemical composition.

The hydrothermal synthesis method is considered to be the most common technique for zeolite synthesis, where the solvent is always water [11]. In solvothermal synthesis, water can be used, but mainly organic solvents are used, including alcohols (methanol, ethanol), ethylene glycol, hydrocarbons, and pyridine [9], while in the ionothermal method, ionic liquids are used, whose main advantage is their low melting point (<100 °C) [11]. Based on the above division of zeolite synthesis methods, it can be seen that all hydrothermal and ionothermal methods are, in principle, included in solvothermal methods, while solvothermal methods are not [32].

### 3.1. Hydrothermal Synthesis

Conventional zeolite synthesis is a time-consuming hydrothermal process carried out in an alkaline environment in the temperature range of 90–150 °C at a pressure of 1–15 bar [6] in a closed system for 24–96 h [17,33]. This involves several steps in which the aluminosilicate hydrogel, organic molecules, and metal cations are converted into crystalline aluminosilicate [34]. Aluminosilicate hydrogels are most commonly obtained from a mixture of compounds containing aluminum (aluminate, aluminum nitrate, and aluminum sulfate) and silicon (water glass, kaolinite, and SO_2_ colloid). Due to the high cost of pure substrates, natural clay materials (e.g., halloysite or kaolin) [9] and waste materials (e.g., fly ash [35], rice husk [36], and paper sludge [37]) are often used as reactants. Crystal nuclei are formed throughout the crystallization process, but the highest formation rate is observed in the initial phase. Hydrothermal synthesis of zeolites at about 100 °C generally results in the formation of crystals between 0.1 and 10 μm [33]. Depending on the transformation parameters used, hydrothermal synthesis can produce different types of zeolite materials, including chabazite, Na-P1, phillipsite, faujasite, or zeolite (Y, X, A, P) [17]. As reported by Johnson and Arshad [32], several key factors must be considered in the hydrothermal synthesis of kaolin-based zeolites as follows:

(a) the Si/Al molar ratio; low (Si/Al ≤ 5) gives SAPO, different types of LTA, and zeolites X, while high (Si/Al ≥ 5) gives beta and ZSM-5 zeolites and different types of zeolite Y;
(b) the appropriate concentration of NaOH (optimum ≤ 3 Mol L^−1^); higher reduces the relative crystallinity and favors the formation of (hydroxy)sodalites as impurities;
(c) the crystallization temperature, which should be between 70 °C and 200 °C; a temperature ≤ 70 °C is not sufficient for the synthesis of crystalline compounds;
(d) the crystallization time (interval < 24 h < 120 h).

Novembre et al. [38] carried out an experiment to obtain Na-X zeolite by a hydrothermal method using natural substrates (naturally zeolitized alkaline volcanic rock and siliceous opaque). The process was carried out at 80 °C using a sodium hydroxide solution at a concentration of 3 Mol L^−1^. The authors showed that the synthesis of the Na-X zeolite started after 5 h and reached the peak of crystallization after 18 h of the process, and the zeolite obtained had a wide (temporal) stability range (500 h).

### 3.2. Various Techniques of Hydrothermal Synthesis

#### 3.2.1. Alkali Fusion

In zeolite synthesis, the fusion method precedes conventional hydrothermal treatment. In this process, the raw material is fused with an alkali (e.g., solid sodium hydroxide), which acts as an activator for zeolitization [34]. The first synthesis step in the process discussed above is the thermal activation of the starting material, which is carried out at temperatures in the range of 500–650 °C [17,39]. This is followed by the aging of the reaction mixture at temperatures between 20 and 50 °C for a period of several to tens of hours [32]. The final step is the crystallization of the reaction mixture, which is typically performed at 100 °C for 24 to 48 h [39,40]. During the fusion process, sodium ions, when the introduced base is sodium hydroxide, act as stabilizers of the crystal structure of the zeolite subunit, increasing the amount of zeolite formed during chemical synthesis [34].

Among others, colloidal silica and sodium silicate are used as siliceous components in the method described above, while aluminum isopropanolate and sodium aluminate are used as zeolitic components [40,41]. The quality of the resulting synthesis product depends on the Na_2_O:SiO_2_:Al_2_O_3_:H_2_O molar ratio, the temperature and activation time, the aging time of the reaction mixture, and the crystallization temperature and time [42]. According to Aylele et al. [43], the advantage of alkaline fusion in the synthesis of zeolite A is the possibility of using low-quality primary kaolin without purification, whereas the conventional hydrothermal method requires high-quality raw material. The suitability of the hydrothermal method preceded by alkaline fusion in the synthesis of Na-A zeolite (Z-S1) from one of the volcanic rocks (scoria) was demonstrated by Lee et al. [44]. Based on their results, the authors concluded that control of the NaOH/precursor ratio is important to ensure high crystallinity of the zeolite product, as well as the size of the particles, which decreases with increasing alkali content in the medium. Thuadaij and Nuntiya [45] also used alkali fusion to obtain Na-X zeolites from fly ash, powdered amorphous silica, metakaolin, and their mixtures. The authors demonstrated that a mixture of metakaolin should be used to produce this type of zeolite and achieved high efficiency in converting mixtures to Na-X zeolite for a SiO_2_/Al_2_O_3_ = 3.25 ratio, where fly ash, amorphous silica, and metakaolin were present in a 1:3:6 ratio.

#### 3.2.2. Alkaline Activation

Synthetic zeolites can be obtained by crystallization in a process known as alkaline activation. This process is mainly used to obtain geopolymers, which are inorganic polymers produced at low temperatures (<100 °C) and consist of chains or networks of mineral molecules linked by covalent bonds [46]. The geopolymers are produced by reacting a low-calcium aluminosilicate, such as silica fly ash, with an alkaline solution [47]. The alkaline activator in this process is a concentrated base, which can be hydroxide, silicate, carbonate, or sulfate [30]. The reactive aluminosilicates are dissolved in an aqueous alkaline solution, then the [SiO_4_]^4−^ and [AlO_4_]^5−^ tetrahedra join corners in a polycondensation process and form subcrystalline or amorphous aluminosilicate space structures with polymeric Si–O–Al–O bonds [48,49]. The process of alkaline activation of aluminosilicate phases contained in fly ash has been described by Garcia-Lodeiro et al. [50]. According to these authors, in materials with significant aluminosilicate content, amorphous hydrated aluminosilicates, zeolites, and gels are formed as a result of alkaline activation. The next reaction products can be zeolites:hydrosodalite, zeolite P, chabazite-Na, and faujasite-Ca. The mechanism of the geopolymerization reaction is not fully understood, and the simultaneous occurrence of stages in the process makes it even more difficult to understand. However, three main stages can be distinguished [51] as follows:

(1) dissolution of silica and alumina in a strong alkaline solution (decomposition of solid aluminosilicates, whose products are a mixture of silicates, aluminosilicates, and aluminates);
(2) diffusion or transport of solutes, polycondensation, and gel formation (condensation reaction of alumina and hydroxylated silica to form the inorganic gel phase of a geopolymer);
(3) hardening of the gel phase—polymerization (formation of a three-dimensional aluminosilicate structure by increasing the connectivity in the geopolymer gel, crosslinking, and reorganization of the network).

Villa et al. [47] synthesized geopolymers by alkaline activation of natural zeolite. They used sodium silicate and sodium hydroxide as activators in proportions of 0.4, 1.5, 5, 10, and 15, using a 7 M sodium hydroxide solution. The time and temperature conditions used during setting and curing were variable, while the activator/precursor ratio was kept constant at 0.6. The results of this experiment showed that increasing the activator/precursor ratio, as well as the curing time, promoted the mechanical strength of the material, with the best results obtained at conditions of 90 days and 40 °C.

Alkaline activation is a polycondensation reaction and leads to the formation of new structures where the resulting negative charge is compensated by monovalent cations (Na^+^ or K^+^) from the alkaline activator (KOH or Na_2_SiO_3_) [3,52]. The combined use of alkali metal silicate with alkali metal hydroxide allows the reaction to occur to a greater extent [3]. Alkali metal cations play a fundamental role in controlling synthesis steps such as curing and crystal formation [53]. Alkali-activated materials are characterized by exceptional mechanical strength, fire and corrosion resistance, durability, rapid curing, and low thermal conductivity. Due to these advantages, the aforementioned materials are mainly used in the construction and thermal insulation industries, but also as catalysts or membranes [3,54].

### 3.3. Molten Salt Method

This method of zeolite synthesis was developed by Park et al. [55]. It involves the reaction of a mixture of NaOH–NaNO_3_ or NaOH–KNO_3_ with fly ash under anhydrous conditions at temperatures above 250 °C [34]. The advantages of this method are the simplicity and versatility of the implementation, the low temperatures, and the favorable cost/purity ratio of the products obtained in one phase. In addition, this method allows the synthesis of zeolites from various types of mineral wastes, has a shorter synthesis time compared to other methods, and does not generate alkaline liquid waste due to the anhydrous conditions [56]. Unfortunately, the lack of water in the environment can lead to insufficient contact between the reactants during the crystallization process, which can reduce the rate of conversion of the precursor to the product and result in an irregular morphological structure of the zeolite [34]. In their experiment, Park et al. [55] used different combinations of salt mixtures on zeolite fly ash, using NaOH, KOH, or NH_4_F as mineralizers and NaNO_3_, KNO_3_, or NH_4_NO_3_ as stabilizers. The reaction mixture contained 0.7 g fly ash, 0.3 g alkali, and 1 g salt, and the whole mixture was heated at 350 °C. The zeolite materials obtained by the authors consisted of sodalite and cancrinite as the main crystalline phases. The suitability of the molten salt method for the synthesis of zeolite materials from sewage sludge was demonstrated by Yoo et al. [57].

### 3.4. Microwave Assisted Synthesis

Zeolites can also be obtained by synthesis using microwave irradiation. This is a simple and effective technique that can reduce the synthesis time of zeolites, improve the homogeneity of their dimensions and composition, and improve the dissolution of the precursor gel [58]. As reported by Panzarella et al. [59], the efficiency of microwave-assisted synthesis is affected by the size of the vessel in which it is performed and the volume of the reaction mixture. For example, microwave heating has been used to obtain zeolite A, ZSM-5, faujazyite, analcime, AIPO_4_-5, and VPI-5 [60,61]. The advantages of synthesizing zeolites using microwave radiation include [61,62]:

(a) much faster heating of the reaction mixture compared to conventional methods,
(b) high reaction efficiency,
(c) ability to control morphology, phase purity, and pore size,
(d) rapid formation of crystallization nuclei,
(e) uniform heating of the entire volume of the reaction mixture.

Anuwattana et al. [63] showed that microwave heating at 150 °C (frequency 2.45 GHz, maximum power up to 1200 W) increased the rate of formation of ZSM-5 zeolite from iron slag by a factor of four compared to hydrothermal heating. It also affected the formation of smaller ZSM-5 particles (0.3 μm vs. 3 μm in diameter). Serrano et al. [64] also demonstrated the usefulness of microwave heating in the synthesis of TS-2 zeolite. The authors demonstrated that it allows a shorter process time, with 100% crystalline samples being obtained after only 15 h, as opposed to the 48 h required by the conventional process.

### 3.5. Other Methods

An interesting method to obtain zeolites is the synthesis inside an inert mesoporous material (confined space synthesis), which is usually carbon. In this preparative technique, the zeolite is crystallized inside the pore system of an inert matrix. In this way, the crystals cannot grow larger than the surrounding pores [65]. The crystals are then separated from the matrix by pyrolysis at 550 °C. The undoubted advantages of confined space synthesis are [66,67] as follows:

(a) high reproducibility,
(b) control of the maximum crystal size by the size of the matrix mesopores,
(c) high purity of the obtained samples,
(d) the possibility of selecting the synthesis conditions to obtain highly crystalline zeolites.

With this method of synthesis, the zeolites obtained are characterized by a well-developed specific surface area and have the same number of acid sites as in the corresponding large zeolite crystals [68].

Another method of obtaining zeolites is the use of dry aluminosilicate gels, amines, and water in the vapor phase (vapor phase transport synthesis, VPT) [69,70]. According to Kim et al. [71], in the first stage of the process, water vapor condenses on the micropores of the precursor, resulting in the establishment of a liquid–vapor equilibrium after some time. The silica present in the gel then reacts with organic cations. The formation of crystal nuclei and the growth of the crystals take place on the outer surface of the precursor. The main influence on crystallization during VPT synthesis is the amount of water in the solvent mixture. The greater the amount of water, the more crystalline and structurally complex the product obtained [70]. The alkalinity of the system also has a significant effect on the crystallization process, determining the rate of this step and the particle size [72]. Liu et al. [73] synthesized MCM-22 zeolite and Niu et al. [72] beta zeolite using this method.

An increasingly popular method of obtaining zeolites is mechanochemical synthesis, in which crude precursors are subjected only to mechanical energy in an environment with little or sometimes no solvent [74]. This method reduces waste, energy consumption, and overhead costs and allows the type, density, and availability of active sites to be influenced by controlled amorphization [75]. Wu et al. [76] synthesized zeolites in the presence of NH_4_F by grinding anhydrous starting solids and heating at 140–240 °C. Under these conditions, they obtained zeolites with MFI, BEA, EUO, and TON structures. The process itself was characterized by a simplified procedure (compared to hydrothermal synthesis) and high efficiency. Ren et al. [77] demonstrated that grinding (10–20 min) of a mixture of chemical reactants (Na_2_SiO_3_·9H_2_O, NH_4_Cl fumed silica, and TPABr) followed by heating at 180 °C for 24–48 h leads to the formation of ZSM-5 type zeolite. The suitability of ball milling in the synthesis of ZSM-5 zeolite and mordenite has also been confirmed by Nada et al. [78]. The obtained zeolites were characterized by a high specific surface area (~300 m^2^ g^−1^), and the whole process took place in the absence of solvents, organic structure directing agents, or grafting crystals.

## 4. Applications of Zeolites

**Figure 4:**
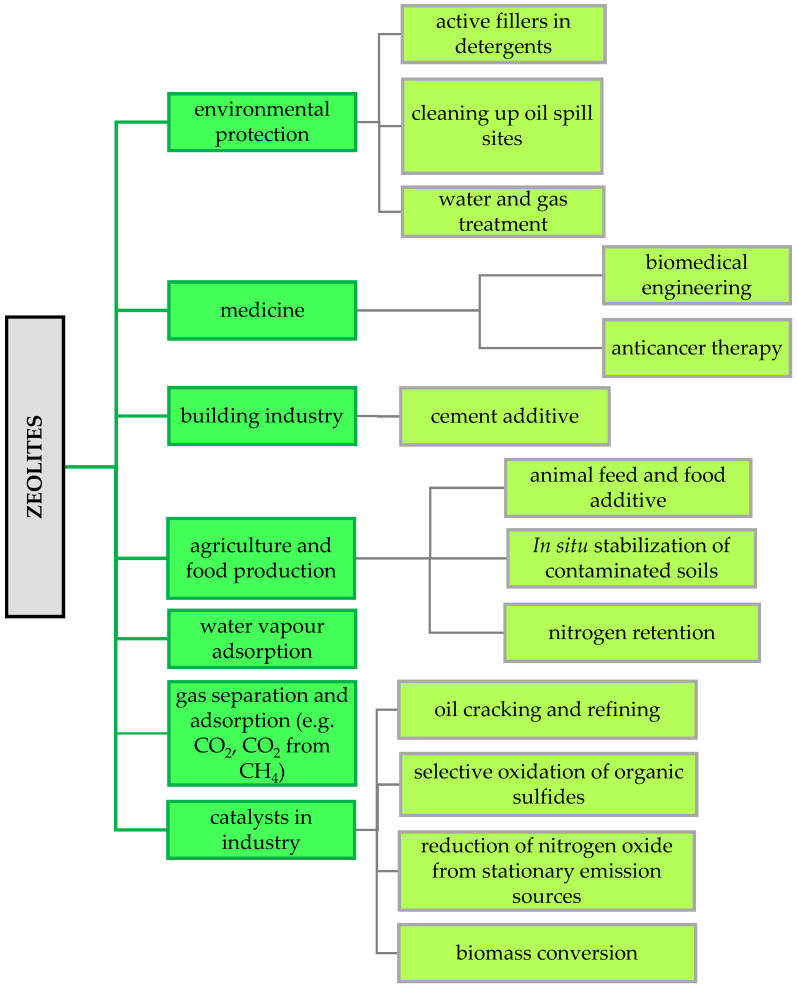
Examples of zeolite applications in industry (own elaboration based on Rhodes [80]).

Zeolites, as microporous materials, have unique physicochemical properties and a unique structure, making them widely used in many modern scientific and industrial fields [79,80] (Figure 4).

The magnitude of the negative charge, compensated by mobile cations, determines the sorption and ion exchange capacity and ion exchange selectivity of the zeolite. Their spatial structure allows for molecular-scale and thread processes and catalytic activity, which can be further enhanced by modifying their surfaces and pores [81]. Due to their unique properties, they are used in catalysis, ion exchange, adsorption, and separation of mixture components [25]. They are also a perspective material in the synthesis of nanostructures, inclusion chemistry, or guest–host complexes [82].

The industrial use of zeolites began in the 1960s, when the US-based Mobil Oil Corporation and Union Carbide used zeolite Y (FAU) as a cracking catalyst in oil processing [83]. A few years later, modified forms of zeolite Y, including rare earth Y (REY) and ultra-stable Y (USY), were obtained in the company’s laboratories, and in 1973, an innovative method for synthesizing high-silicon zeolite ZSM-5 using organic alkylammonium cations as crystallization guides was developed [83]. In the following years, a number of new and modified zeolites were synthesized and found wide applications in industrial processes related to adsorption, ion exchange, and catalysis [11].

Hierarchical (or mesoporous) zeolites are of increasing interest. They are obtained by top–down (desilication, dealumination, recrystallization, and irradiation) and bottom-up (template-free, soft templating, hard templating, double templating with surfactant, nanoparticle assembly, and zeolization of materials) methods [84]. However, the most efficient and cost-effective way to synthesize hierarchical zeolites is the desilication process, which involves the preferential removal of silicon from the zeolite structure by OH^−^ ions in an alkaline medium (usually a NaOH solution) [85]. The obtained product is characterized by the presence of a secondary mesopore system within each grain (which ensures a relatively free diffusion of reactants to and from the active centers and the transport of branched molecules) while maintaining its microporous nature and high-performance acid centers [84]. The best-studied technique for removing silicon from the zeolite skeleton is treatment with NaOH solution (0.2 Mol L^−1^) at 65 °C for 30 min at a ratio of 1 g of zeolite per 30 mL of solution [86]. Hierarchical zeolites have been used primarily as catalysts in reactions such as cracking, hydrocracking, alkylation, or isomerization [87].

### 4.1. Zeolite Applications in Agriculture

Worldwide, agriculture is the main user of natural zeolites. However, in the agricultural sector, zeolites are mainly used in animal husbandry (bedding and feed additives), and about 30% of the mineral is used as a soil additive [88].

#### 4.1.1. Soil Amendment with Multidirectional Action

Zeolites are considered to be one of the most widely used natural inorganic agents to improve the physical and chemical properties of soils [89]. The presence of large pores in zeolites allows them to retain water in their structures [29], and thus, due to their unique properties, zeolites can increase water use efficiency (WUE) by increasing the water holding capacity (WHC) of the soil [90]. Xiubin and Zhanbin [91] showed that the WHC of zeolite-treated soil increased by 0.4–1.8% under drought conditions and by 5–15% under normal conditions compared to the control soil. According to the authors, zeolite application can reduce surface runoff and protect the soil from erosion, as well as regulate crop water supply under severe drought conditions. Zeolites also improve infiltration rate and saturated hydraulic conductivity [92], cation exchange capacity (CEC) [93], water-holding capacity, and aeration [28,89]. Soils with zeolites can, therefore, better retain rain and snowmelt water and prevent it from percolating deep into the soil profile (beyond the root zone) [89]. Zeolites have a positive effect on the geometric properties of soils, including specific surface area and porosity [88]. An increase in specific surface area and a decrease in pore size can result in, among other things, reduced oxygen diffusion, mineralization of humic compounds, and loss of organic carbon stocks. This phenomenon is particularly beneficial in soils with low organic matter content and relatively poor aeration [94]. Zeolite incorporation can also improve nutrient retention [14] and help buffer soil pH, reducing the need for lime [28]. Bikkinina et al. [95] demonstrated in a field experiment that the application of zeolites to leached black soil resulted in an increase in soil pH, plant-available phosphorus and potassium, improved microbial activity in the rhizosphere, and accelerated microbial biomass growth. Due to its alkaline nature and the presence of a negative charge, phosphorus availability is increased in zeolite-enriched soils following an increase in soil pH and a decrease in the amount of exchangeable iron and aluminum ions [90,96]. The specific physicochemical properties of zeolites make them capable of releasing nutrients gradually, increasing productivity and efficiency of fertilizer use, reducing losses, and thus reducing environmental pollution [97].

By reducing the intensity of the nitrification process, the addition of zeolites reduces the risk of nitrate leaching into groundwater [98]. Zeolites have been shown to have a particularly high affinity for NH_4_^+^ [99,100]. The presence of small pores (nominal pore size 4–5 Å) in the structure of the zeolite crystal lattice, in which ammonium cations are easily adsorbed, makes them unavailable to nitrifying microorganisms and conversion to NO_3_^+^ [90]. Thus, in zeolite-treated soils, there is improved retention of this cation and slower release into the soil substrate, which increases the efficiency of its utilization and improves crop yields [101]. Ahmed et al. [102] showed that the application of inorganic fertilizers mixed with zeolites significantly increased the uptake of nitrogen, potassium, and phosphorus and their application efficiency in maize crops. Similar observations were made by Li et al. [103], who found an increase in spinach yield and plant nutrient assimilation in a greenhouse experiment after the combined application of zeolite, ammonium, and potassium.

#### 4.1.2. Crop Protection

The ion exchange capacity and potentially high sorption capacity of zeolites can also be successfully exploited when used as carriers for pesticides and herbicides [14]. Shirvani et al. [104] conducted a study to develop slow-release formulations (SRFs) of 2,4-dichlorophenoxyacetic acid (2,4-D) using, among others, zeolite modified with cetyltrimethylammonium bromide (CTAB) as a surfactant. The authors showed that the SRF had the same herbicidal efficacy as free (technical) 2,4-D. In addition, it significantly reduced the mobility of the herbicide in the soil and reduced its desorption. After 168 h, between 62% and 64% of the adsorbed 2,4-D was released into the solution phase. According to the researchers, the SFR can be considered an effective tool for weed control in sustainable agriculture. The formulations release the active ingredients of the herbicides gradually, reducing their loss through leaching and biodegradation, thereby reducing the negative environmental impact of herbicides. Similar conclusions were reached by Bakhtiary et al. [105].

Another use of zeolites in agriculture is as plant protection products against pests and fungal diseases. Calzarano et al. [106] used a spray of crushed zeolite (15 kg L ha^−1^) to control gray mold and sour rot in a white grapevine variety. The researchers showed that this strategy was effective, reducing the risk of infection by more than 70% for both diseases. The antifungal effect of zeolite is based on the formation of a layer of mineral particles on the treated plant, which forms a physical barrier that inhibits the germination and development of acid rot and gray mold fungi. According to the authors, reflectance measurements performed on the leaves of the treated grapevines showed no differences compared to the control series in terms of NDVI (Normalized Difference Vegetation Index) and GNDVI (Green Normalized Difference Vegetation Index), whose values correlate with the amount of biomass and chlorophyll content. A similar study was carried out by Prisa [107], which confirmed the usefulness of micronized zeolite as a fungicide in viticulture. Its foliar application effectively reduced the development of diseases caused by *Botrytis cinerea*, *Oidium tuckeri*, and powdery mildew compared to the application of copper and sulfur. At the same time, zeolite had a positive effect on the vegetative and root growth of *Vitis vinifera* and showed no phytotoxicity. In his opinion, zeolite is an ecological and cost-effective tool for increasing plant productivity in sustainable agriculture.

#### 4.1.3. Heat Stress and Photosynthesis Enhancement on Crops

Another advantage of the foliar application of zeolites is the increase in carbon dioxide near the stomata and the reduction in leaf temperature by reflecting infrared radiation [108]. Zeolites are able to adsorb carbon dioxide molecules and release them gradually into the ecosystem [109], which in turn can increase the photosynthetic rate of C3 plants [108], such as grapevines, tomatoes, apple, and orange trees [14]. This results in increased vegetative growth [110], an increased leaf area production rate, and a reduced transpiration rate [111].

#### 4.1.4. Aquaculture

In aquaculture, zeolites are used to reduce the amount of algae in water reservoirs or farm ponds, assist in the elimination of ammonia from water [112], and are also used to aerate aquatic organisms with oxygen produced by air separation [14]. The addition of zeolites to fish ponds reduces turbidity, with positive effects on water quality, fish health, and growth performance [113].

### 4.2. Zeolites in Environmental Protection

The use of zeolites in environmental protection is mainly based on their ion exchange properties [114]. Other properties that determine their suitability in this field are their significant adsorption capacity, long-term mechanical and physical stability, and strong selectivity and molecular sorption capacity [11,115].

#### 4.2.1. Sorption of Radionuclides

Zeolites are widely used to sequester cationic contaminants such as the trace elements lead, cadmium, zinc, nickel, manganese, chromium, copper, and iron [90]. They are also used to extinguish chemical fires and to deactivate nuclear and other hazardous industrial wastes [114]. A study by Osmanlioglu [116] evaluated the usefulness of clinoptilolite in the removal of radionuclides (^137^Cs, ^60^Co, ^90^Sr, and ^110^Ag) from liquid radioactive waste. It was found to be an effective sorbent of radionuclides under dynamic processing conditions and can be used as a cheaper alternative to chemicals in the chemical precipitation process. A limitation in the use of clinoptilolite is the high content of inactive salts in the radioactive waste, which reduces the ion exchange capacity of zeolite towards ^90^Sr and ^60^Co.

Lihareva et al. [117] also demonstrated the usefulness of clinoptilolite for the removal of Cs^+^ and Sr^+^ from aqueous solutions, reporting a maximum adsorption capacity of 122.7 and 21.50 mg g^−1^, respectively. Borai et al. [118] evaluated four different zeolite minerals (natural clinoptilolite, chabazite, mordenite, and synthetic mordenite) for their utility in removing certain radionuclides from low-level radioactive liquid waste (LLRLW). They demonstrated that of the materials tested, natural chabazite had the highest decay rates and Cs ion exchange capacity. Promising results for the removal of radium isotopes from mine water using zeolite NaP1 were obtained by Chałupnik et al. [119]. The water purification efficiency exceeded 98% for the radium isotopes ^226^Ra and ^228^Ra. In addition, they confirmed the possibility of removing radium from very saline waters (TSD > 100 g L^−1^) using the zeolite material.

#### 4.2.2. Immobilization of Trace Elements in the Soil

Zeolite remediation of contaminated soils reduces the amount of phytoavailable forms of trace elements, leading to the restoration of soil homeostasis [120,121]. When mixed with Portland cement, zeolites are an effective stabilizing agent and, in the case of trace elements, an immobilizing agent [122]. The mechanism of trace element adsorption using zeolites includes the following phenomena: (1) ion exchange; (2) electrostatic attraction; (3) intrapore adsorption; (4) surface complexation; and (5) surface precipitation [123]. The pH value of the solution has an important influence on the above-mentioned processes, as it affects the surface charge of the zeolite and, consequently, the adsorption of trace elements [124]. The presence of free cations in the zeolite skeleton allows ion exchange with cations present in solution (mainly off-grid sodium participates in the exchange process) [125]. The effect of zeolites as contaminant sorbents is to bind harmful trace elements into insoluble compounds or organic–mineral complexes [126], which are less available to plants and immobilized in the soil in a safe form for a long time [127]. This is confirmed by our study [128], in which the application of a molecular sieve (crystalline aluminosilicate with a micropore size of 0.3 nm) reduced the concentration of iron (by 5%), nickel (by 8%), cadmium (by 18%), chromium (by 22%), zinc (by 22%), copper (by 13%), and manganese (by 44%) in the aerial parts of sunflowers grown in copper-contaminated soil compared to the control. In contrast, in the roots of sunflowers, zeolite application to soil contributed to a decrease in chromium and zinc content by 15% and 4%, respectively. The beneficial effect of zeolite application on the immobilization of trace elements in contaminated soil was also demonstrated by Cadar et al. [79]. They showed that a 10% addition of this material to the soil reduced the bioaccumulation of Co, Cr, Cu, Mn, Ni, Pb, and Zn in the roots and shoots of spinach, parsley, and lettuce, with the exception of Cd in the spinach roots. Zeolite had an analogous effect on the content of nickel and copper in oat roots [129]. Li et al. [130] also confirmed the usefulness of zeolite in the remediation of lead-contaminated garden soil. The addition of natural zeolite (20 g kg^−1^) increased soil pH, exchange capacity, and organic matter content and facilitated the formation of soil aggregates. It also reduced the bioavailability of lead and its uptake by canola. At the highest level of soil contamination with lead (2000 mg kg^−1^), the content of the analyzed element in plant roots decreased by 49% and in shoots by 30% compared to the control series. In a study by Wyszkowski and Brodowska [131], zeolite reduced the content of zinc, manganese, and cobalt, and in the experiment by Kosiorek and Wyszkowski [132], it reduced the content of copper and nickel in maize. The application of zeolite to soil contaminated with trace elements also affects the structure and microbial activity by increasing the activity of the enzyme dehydrogenase, thus improving soil condition and fertility [133].

#### 4.2.3. Gas Adsorption and Catalysis

All natural and synthetic zeolites can be used for the selective adsorption of components of gas mixtures, their drying and purification, and odor control due to the variation in pore size and the presence of cations in the structure [14]. They are used in intensive livestock farming to reduce odors caused by H_2_S and NH_3_ [134,135]. They also reduce the humidity in such areas [14]. The ammonium adsorption capacity of zeolites ranges from 8.149 mg N g^−1^ to 15.169 mg N g^−1^ [136]. In addition, zeolite can be widely used in combination with other additives to reduce gas emissions, salinity, and nutrient loss during the composting process [137,138]. Wang et al. [139] evaluated the effect of adding zeolite, wood vinegar, and biocarbon on the composting process of pig manure. After 50 days, the authors reported a reduction in methane (by 50.39–61.15%), carbon dioxide (by 33.90–46.98%), and nitrous oxide (by 79.51–81.10%), and a reduction in ammonia loss (by 64.45–74.32%) compared to the control (no additives). The positive effect of zeolite addition on the composting process of dewatered fresh sludge has also been demonstrated by Awasthi et al. [137]. The best results were observed in the series with a 30% zeolite and 1% lime addition. This treatment significantly reduced ammonia, methane, nitrous oxide emissions, and nitrogen losses (by 50%) compared to the control series. The addition of zeolite increased the initial pH, had an activating effect on the total aerobic bacterial population, and increased the porosity of the feedstock and the composting rate. Under these conditions, compost maturity was achieved in 37% less time (35 days versus 56 days) than in the control sample.

The specific size of the channels inside the structure of zeolites allows them to act as molecular sieves and selectively adsorb components of gaseous mixtures. In addition, physicochemical modification of zeolites makes it possible to obtain materials with desired properties. Akyalcin et al. [140] conducted an experiment to develop a method for obtaining hydrogen (H_2_) storage materials. The authors used clinoptilolite, which they treated with various chemical compounds (HCl, C_2_H_2_O_4_, and HNO_3_). The authors evaluated the effects of the applied solution concentration (0.1–1.0 Mol L^−1^), temperature (60–80 °C), and treatment time (2–4 h) on the hydrogen adsorption capacity of the zeolite. The best results were observed after treatment with 0.5 Mol L^−1^ HNO_3_ at 80 °C for 2 h. Clinoptilolite modified under these conditions exhibited a 7.3-fold higher H_2_ adsorption capacity relative to the crude material. The better H_2_ adsorption capacity of the modified clinoptilolite was associated with an increase in the zeolite’s specific surface area, volume, and size of micropores, as well as an increase in the strength of the acid centers.

The catalytic properties of zeolites are related to their unique properties with respect to the specific surface area, pore size (shape-selective catalysis), crystallinity, and thermal stability. In addition, the presence of proton donor (bridged -OH; Brønsted acid centers), electron acceptor (Al cross-linked tri-correlated; Lewis acid centers), and electrodonor (O_2_^−^ and AlO_4_^2−^; Lewis base (alkali) centers) groups enables zeolites to catalyze many reactions on an industrial scale [25]. Clinoptilolite and mordenite are used as adsorber catalysts for the removal of SO_2_ from gas and flue gas streams from factory stacks [141]. Zeolites are also used in environmental catalysis as adsorbents and catalysts for the reduction of nitrogen oxides (NO_x_) and volatile organic compounds (VOCs) [142]. The aforementioned compounds are major air pollutants, and their source is the combustion of fossil fuels, both stationary (power plants) and mobile (automobiles) [143], as well as metallurgy [142] and nitric acid plants [144]. Most techniques to reduce the formed nitrogen oxides involve the introduction of a reductant (e.g., ammonia or urea) into the waste gas and its reduction to molecular nitrogen and water [145,146]. Due to its high efficiency and wide temperature window (150–450 °C), the most widely used NO_x_ abatement technology is selective catalytic reduction using ammonia as the reducing agent (NH_3_-SCR) [147]. A commonly used commercial catalyst in the NH_3_-SCR process is vanadium oxide supported on titanium oxide (V_2_O_5_-TiO_2_) [148]. However, due to its unsatisfactory efficiency and the toxicity of vanadium compounds to the environment [144], alternative catalysts are being sought for the NH_3_-SCR process. Zeolite systems doped with transition metal ions (mainly copper and iron) are promising [149].

The characteristics of zeolites that support their usefulness in the deNO_x_ (NO_x_ destruction) process are mainly high catalytic activity, favorable temperature window, high thermostability, chemical resistance [150,151], and hydrothermal stability [152]. Zeolites of great interest for application in the deNO_x_ process include zeolite ZSM-5 [153,154], SAPO-34 [155], Fe-Beta [156], or iron-modified clinoptilolite (after its application, the NO_x_ conversion rate exceeded 90%) [144]. Iron-enriched zeolite catalysts are mainly active at medium, and high temperatures [150]. An example is the Fe-ZSM-5 zeolite, which shows high activity in the temperature range of 300 °C to 450 °C [157]. However, at such high temperatures, carbon deposition can occur and slow down the process [158]. Consequently, copper-based zeolite catalysts, which are highly active at lower temperatures (<300 °C) despite their lower hydrothermal stability, are of greater interest [150,159]. The deposition of copper in the zeolite support (in the amount of 2–4 wt%) increases the deNO_x_ efficiency up to 95% [158]. The Cu-SSZ-13 catalyst has found commercial application in diesel engines to reduce nitrogen oxides in the exhaust [160]. Paolucci et al. [161] presented the mechanism of the deNO_x_ process on Cu-SSZ-34, paying particular attention to the redox cycle of copper forms that change their degree of oxidation: CuII ↔ CuI. The desired N_2_ product is formed within two half-cycles (reduction and oxidation), which is a new insight into the mechanism of the SCR reaction. Ammonia as a reductant in the SCR process can be replaced by methane, which has an inert chemical character and lower cost [162]. Cobalt-ion-supported zeolite-based systems catalyze the selective NO reduction reaction with methane in the presence of water [163,164]. The addition of a second metal, such as palladium or platinum, favorably affects their catalytic properties and improves the efficiency of the reduction process [165,166]. Cobalt-enriched zeolites alone exhibit high NO_x_ reduction activity and nearly 100% reduction to nitrogen at temperatures above 400 °C [167,168]. In contrast, the ZSM-5 zeolite based on cobalt, platinum, and nickel shows high activity in the catalytic oxidation of VOCs such as benzene and toluene to CO_2_ and H_2_O [169].

In terms of energy and heating solutions, zeolites can serve as components of heat exchangers [170]. They are used to store heat from solar radiation, off-peak electricity, or waste heat and then use this energy in air temperature control or water heating processes [171].

#### 4.2.4. Wastewater Treatment

In wastewater treatment technologies, zeolites are mainly used for the removal of biogenic compounds (nitrogen and phosphorus), radioactive elements, and trace elements [172]. Natural zeolites allow the removal of ammonium nitrogen from wastewater in amounts ranging from 0.4 to 25.5 mg g^−1^ adsorbent [173,174]. According to Huang et al. [175], the equilibrium state of ammonium ion sorption on zeolites is reached after about 60–120 min. The following parameters influence the efficiency of the process: the contact time of the wastewater with the zeolites, the pH of the solution, the dose and type of zeolites, and the presence of other ions in the solution [172]. Modification of zeolite surfaces with alkalis and strong acids improves the sorption capacity of the mineral for cations [90]. Liang and Ni [176] carried out a modification of a natural zeolite (55% was clinoptilolite) to increase the absorption of ammonium ions. The modification included pretreatment (grinding and sieving), treatment with 1.5 Mol L^−1^ sodium chloride (NaCl), and calcination. After these processes, the specific surface area, total pore volume, and average pore size of the initial zeolite increased. The authors noted the most favorable effects after the combined use of NaCl modification and calcination. Such treatment increased the temperature resistance of the zeolite (from 150 °C to 400 °C) and also increased the ammonium ion uptake rate (AIU) by 4.3 times compared to the raw zeolite. The effect of clinoptilolite modification with ultrasound-assisted chemicals (NaOH, HCl, and FeCl_3_) on the efficiency of ammonium ion removal from water was evaluated by Jahani et al. [177]. The highest ammonium removal efficiency (≥99%) was observed after the acid-modified zeolite. In comparison, the original zeolite removed the contaminant with an efficiency of 51.66%. Importantly, the authors used the modified zeolite five times in the ammonium removal process, and it showed stability in terms of its structural and adsorption properties.

Clinoptilolite shows high selectivity towards heavy metal cations in the following order: Pb^2+^ > Cd^2+^ > Cu^2+^ > Co^2+^ > Cr^3+^ > Zn^2+^ > Ni^2+^ > Hg^2+^ [178]. For this reason, Galletti et al. [179] conducted an experiment to evaluate the suitability of clinoptilolite as a low-cost adsorbent for the removal of Zn^2+^ and Cd^2+^ ions from wastewater in a batch system. Complete adsorption for both analyzed ions was achieved by the authors at a solution pH of 4.5 and a sorbent concentration of 10 mg L^−1^. Interestingly, in the presence of both ions, clinoptilolite showed a higher affinity towards Zn^2+^ than towards Cd^2+^, i.e., the opposite of the single system. This was probably due to a higher partition coefficient for zinc than for cadmium and a stronger binding of the zeolite to zinc. In contrast, Senila et al. [180] showed that clinoptilolite, in addition to being an adsorbent of contaminants, can also act as a carrier for biofilm formation and microbial growth involved in biological wastewater treatment, which has a beneficial effect on the overall process. The usefulness of silver-modified clinoptilolite in immobilizing Cr(VI) ions from model wastewater was demonstrated by Panayotova [181]. In this study, the effect of the reaction (pH 4, 6, and 8) and initial Cr(VI) ion concentration (10 and 20 mg L^−1^) on the removal efficiency of the analyzed pollutant was evaluated. The studies were conducted at a constant ratio of v:m = 100, i.e., 100 mL of wastewater per 1 g of zeolite. It was shown that Cr(VI) immobilization increased with increasing pH values. The best results were obtained after 45 min at pH 8 and an initial Cr(VI) concentration of 20 mg L^−1^. Under these conditions, 82.4% of the chromium ions were removed from the model solution. In the case of industrial wastewater, the analyzed zeolite allowed a reduction of Cr(VI), Cu(II), and Zn(II) contents of more than 80%, 75%, and 70%, respectively, within 30 min. Zeolites can also be used to remove phosphorus compounds from wastewater, but only at an acidic pH. Under these conditions, sites on the zeolite surface become protonated, acquiring a positive electrical charge and attracting phosphate ions. A chemical interaction then takes place between the zeolite surface and the phosphate molecules, which are immobilized [6]. This is confirmed by the studies of Zhang et al. [182] and Goscianska et al. [183].

Table 3 summarizes the directions of application of selected natural and synthetic zeolites.

The application of a particular type of zeolite in a particular field is determined by the properties it exhibits. These include the chemical nature of the surface, the strength of the active sites, the porous structure, the number, type, and distribution of pores, or the molar ratio of Si/Al. For example, Garshasbi et al. [187] used synthetic zeolite 13X in their study of H_2_S separation from a butane gas mixture because of its high operating efficiency at low partial pressures of the recovered components and high selectivity at temperatures up to 100 °C. In addition, it tends to adsorb smaller molecules due to its uniform pore entrance diameter. In contrast, Hashemi et al. [189] used a synthetic faujasite or zeolite Y to remove organic pollutants from wastewater, mainly because of its structural stability and large available pore volume, as well as its Si/Al ratio of ≥1.5, which increases its affinity for non-polar substances.

Clinoptilolite is the most abundant natural zeolite, forming extensive and abundant deposits throughout the world. It has a two-dimensional structure of 8-ring and 10-ring channels [116], and the Si/Al ratio is greater than 4, which makes it characterized by the high thermal stability of the structure [140]. It is also characterized by a high ion exchange capacity and a particular affinity for heavy metal cations. It can adsorb elements such as ^137^Cs, ^90^Sr, and other radioactive isotopes from solution and hold them in its three-dimensional crystal structure [117]. The low cost of clinoptilolite makes its use in the treatment of radioactive waste very attractive [116]. In addition, synthetic zeolites are produced by hydrothermal methods using fly ash as a raw material, which may contain elevated levels of radionuclides. This, in turn, requires monitoring of the natural radioactivity of synthetic zeolites during their synthesis [119]. The aforementioned properties of clinoptilolite support its superiority in the application of radionuclide sorption processes, as confirmed in their studies by Osmanlioglu [116] or Lihareva et al. [117].

Clinoptilolite contains the exchangeable cations Na^+^, K^+^, Ca^2+,^ and Mg^2+^ [140], which allow ion exchange and are the basis of many processes, including drinking water treatment. These properties of zeolite, as well as economic considerations, determined the use of this natural zeolite in a study by Erdem et al. [188] to remove Co^2+^, Cu^2+^, Zn^2+^, and Mn^2+^ ions from industrial wastewater.

In an experiment carried out by Goscianska et al. [183], zeolites Na-P1 and Na-A were used to remove phosphorus compounds from wastewater, since synthetic zeolites obtained from fly ash have a high cation exchange capacity and are able to capture phosphates from solution in oxyanionic forms.

### 4.3. Other Applications of Zeolites

As mentioned earlier in this paper, zeolites are mainly used in traditional industries, such as catalysts in the petroleum industry, molecular sieves, adsorbents in environmental protection, or soil additives in agriculture. Their innovative and future applications seem to be in medicine and biotechnology. According to Pavelić and Hadžija [196], natural and synthetic zeolites have great potential for biomedical applications and can, therefore, contribute to significant advances in the pharmaceutical industry and biology.

Zeolites have found medical applications in modern drug delivery systems [197], wound healing [198], hemodialysis [199], and tooth root canal filling [200]. The adsorption and ion exchange properties of natural clinoptilolite have been exploited in the development of an anti-diarrheal drug [201] and a gastric acid neutralizer [202]. The aforementioned chewable tablets proved effective in the treatment of people suffering from hyperacidity due to gastric dyspepsia and gastric and duodenal ulcers. They had no side effects, did not alter the structure of pepsin, and their physical and chemical properties remained stable after three years of storage at room temperature [202].

#### 4.3.1. Adsorption of Harmful Substances

Due to their detoxifying properties, zeolites are used as materials to remove harmful substances such as pesticides, mycotoxins, or heavy metals. The neuroprotective potential of clinoptilolite in mice exposed to Pb^2+^ was demonstrated by Basha et al. [203]. The experiment consisted of intraperitoneal administration of lead acetate (100 mg kg bw^−1^ day^−1^) to three-week-old mice for 21 days, followed by combined treatment with EDTA and clinoptilolite (100 mg kg bw^−1^) for 2 weeks. The authors observed that this contributed to a reduction in lipid peroxidation and the induction of antioxidant mechanisms, as manifested by an increase in catalase, superoxide dismutase, glutathione peroxidase, and glutathione activity.

#### 4.3.2. Tissue Engineering

Zeolites are also used in tissue engineering [204], in the formation of implant coatings, and in the preparation of fungicidal dressings and antibacterial agents [194]. Their porous nature and high biocompatibility make them ideal materials for bone tissue cell adhesion and proliferation [205]. The high osteogenic potential of zeolite materials and the ability to customize pore shape and size allow the creation of diverse scaffolds with a wide range of biomedical applications. As implant coatings, zeolites increase bone conductivity, aid in local elastic modeling, and reduce local inflammation [204]. As reported by Banu et al. [206], zeolites may also help prevent postmenopausal bone loss. In their experiment, Bedi et al. [195] prepared biocompatible zeolite coatings for use in biomedical implants. Synthesized on commercially pure titanium and Ti_6_Al_4_V alloys, the MFI zeolite coating had higher corrosion resistance than the titanium alloy and reduced the release of cytotoxic Al and V ions into the surrounding tissue. In addition, it had excellent adhesion to the substrate, which could potentially prevent implant loosening. The tested coating also reduced the mismatch between the module and the bone tissue and had a positive effect on implant osteointegration. The aforementioned benefits of MFI zeolite coating indicate its potential in dental and orthopedic applications to facilitate patient recovery after surgery.

#### 4.3.3. Carriers of Bioactive Compounds

Zeolites, due to their high specific surface area, stability, and susceptibility to modification, provide encapsulation of biologically active substances and their controlled release, making them an excellent biomaterial for drug delivery systems [207]. Linares et al. [208] conducted a study to use zeolite cancrinite as a carrier for acetylsalicylic acid and to determine the hydrolytic stability of the drug. They demonstrated that it is possible to administer zeolite as an anti-acid drug and an acetylsalicylic acid carrier at the same time, since no loss of individual pharmaceutical effect was observed for either substance analyzed. In an experiment carried out by Arruebo et al. [193], nanocomposites of magnetite and commercial Na-Y zeolite were formed by mechanical activation during high-energy milling at room temperature. They were characterized by a high specific surface area (442.9 m^2^ g^−1^) and cationic capacity, which allowed the adsorption, storage, and release of significant amounts of doxorubicin, an antibiotic widely used in cancer chemotherapy. Such a drug carrier can be directed directly to the tumor cells and released upon application of an external or internal magnetic field. In this way, the therapeutic dose used to date can be reduced, and the side effects associated with drug application can be reduced [209].

Zeolites can also be used as carriers for substances with antimicrobial properties. Neidrauer et al. [194] evaluated the usefulness of an antimicrobial ointment for the treatment of acute and chronic wounds, in which the active ingredient was nitric oxide embedded in zeolite A. The researchers observed that the zeolite, when subjected to ion exchange with zinc ions and loaded with nitric oxide, gradually released it over 3 h after contact with water in the skin. The minimum microbicidal concentrations (MMC) of the tested ointment against bacterial organisms (5 × 10^7^ c.f.u.) ranged from 50 to 100 mg, while against the yeast *C. albicans* (5 × 104 c.f.u.), the MMC was 50 mg. After 8 h of exposure to zeolite ointment, a reduction in bacterial (by 5–8 log cycles) and fungal (by 3 log cycles) cell viability was observed compared to the control series. In addition to its therapeutic effect, zeolite also promotes the healing process of bacterially infected wounds. The mechanism of antimicrobial action of zeolites is not fully understood but is probably based on physical adsorption, ion exchange, and indirect catalysis [210]. Due to their physical adsorption capacity, zeolites immobilize microbial cells on their surface, leading to their death [211]. In contrast, the chemical interaction of zeolites with microorganisms involves the release of positively charged metal ions (e.g., copper and silver) [212] or reactive oxygen species (e.g., hydrogen peroxide) from their structures into the microenvironment [213], resulting in damage to microbial cell walls and membranes, loss of membrane potential, and ultimately cell destruction and death.

Zeolites, therefore, have many applications. Applications related to the reduction in agriculture and environmental pollution appear to be particularly interesting.

## 5. Conclusions

The popularity of zeolites has been growing steadily since their first industrial applications (second half of the 20th century) until the present day, and research into their properties, synthesis methods, and directions of use has not lost its relevance. This is evidenced by the ever-increasing number of known zeolite structures described in the IZA (International Zeolite Association) database. This environmentally and economically friendly material remains a challenge for science, contributing to the further development of highly active catalysts, adsorbents, and ion exchangers with high selectivity.

The development of methods for the chemical synthesis of zeolites and the modification of their surface has led to materials with new porous structures and physical properties, contributing to almost unlimited possibilities for their commercial and environmental applications.

Zeolites remain a material of the future. Increasing industrialization, climate change, and many years of anthropopressure are expected to be the main factors influencing the development of the market for the aforementioned materials due to the potential for the use of zeolites in environmental protection and agriculture, as well as the growing demand for innovative materials in biomedical processes, active substance delivery systems, and other applications.

## Figures and Tables

**Table 1:** Classification of zeolites based on the size of pores in the structure (own elaboration based on Mijailović et al. [13]; Kulprathipanja [21]).

Type of Zeolite	Membered Rings (MR)	Pore Diameter [nm]	Example of Zeolite
With small pore size	8	0.3–0.45	zeolite A
With medium pore size	10	0.45–0.6	ZSM-5, MCM 22
With large pore size	12	0.6–0.8	zeolite X, Y
With very large pore size and zeolite-like materials	14	0.8–1.0	UTD 1 (14 MR) VIP 5 (18 MR) Cloverite (20 MR)

**Table 3:** Examples of practical applications of selected zeolites in various industries.

Kind of Application	Zeolite Type	Reference
Removal of radionuclides (^137^Cs, ^60^Co, ^90^Sr, and ^110^Ag) from liquid radioactive waste by clinoptilolite	clinoptilolite	[116]
Removal of radium isotopes from mine water	Na-P1	[119]
Catalyst-adsorbent for fuel oil desulfurization	faujasite	[184]
Adsorption of NH_3_	faujasite	[185]
Selective catalytic reduction of NO_x_ with ammonia	ZSM-5	[153]
Catalytic decomposition of NO_x_	SAPO-34	[155]
Adsorption separation of CO_2_/CH_4_ (e.g., biogas upgrading)	zeolite 5A	[186]
Separation of H_2_S from Butane Gas Mixture	13X	[187]
Industrial wastewater treatment (removal of Co^2+^, Cu^2+^, Zn^2+^, Mn^2+^)	clinoptilolite	[188]
Removal of organic pollutants (including toluene, styrene, hexadecane, octadecane) from wastewater	zeolite Y	[189]
Removal of phosphorus compounds from wastewater	Na-P1 and Na-A	[183]
Aromatic alkylation (petrochemical industry)	MCM-22	[190]
Dewaxing catalysts for hydrocarbon feeds	SAPO-11, ZSM-23	[191]
Trace element immobilization in soil	clinoptilolite	[79]
Reduction of NO_3_ leaching from soil and optimization of plant growth	chabasite	[97]
Buffering soil pH, increasing cation exchange capacity (CEC)	clinoptilolite	[88]
Increasing soil water holding capacity and infiltration rate of mordenite	mordenite	[91]
Slow Release of Herbicides	zeolite Y	[192]
Retention of nutrients (N, P, and K)	clinoptilolite	[102]
Drug Delivery System (DDS) (antibiotic)	Na-Y	[193]
Drug Delivery System (DDS) (NO, antibacterial)	zeolite A	[194]
Bone tissue engineering	ZSM-5	[195]

## Data Availability

All data are available in the manuscripts and from the authors.
